# Interaction of phenanthrene and potassium uptake by wheat roots: a mechanistic model

**DOI:** 10.1186/1471-2229-13-168

**Published:** 2013-10-26

**Authors:** Xinhua Zhan, Xiao Liang, Tinghui Jiang, Guohua Xu

**Affiliations:** 1 College of Resources and Environmental Sciences, Nanjing Agricultural University, Nanjing, Jiangsu Province 210095, P.R. China; 2 State Key Laboratory of Soil and Sustainable Agriculture, Institute of Soil Science, Chinese Academy of Sciences, Nanjing, Jiangsu Province 210008, P.R. China

**Keywords:** Phenanthrene, Plasma membrane H^+^-ATPase, Polycyclic aromatic hydrocarbons, Potassium, Wheat root uptake

## Abstract

**Background:**

Polycyclic aromatic hydrocarbons (PAHs) are potentially carcinogenic, mutagenic and toxic to both human and non-human organisms. Dietary intake of PAHs is a dominant route of exposure for the general population where food crops are a major source of dietary PAHs. Over 20% of main food crops contain PAHs that exceed the control limits in China. However, the mechanisms on PAH accumulation in crops are not well understood.

**Results:**

Here we report the physiological mechanism of potassium (K^+^)-stimulated uptake of phenanthrene (PHE, a model PAH) in wheat. PHE uptake is stimulated by the external K^+^. The addition of blockers (tetraethlyammonium and barium) for K^+^ channels does not suppress the process, suggesting that K^+^ channels are not involved. The introduction of PHE and K^+^ elicits a much greater depolarization in root cell membrane potential than that of either PHE or K^+^. K^+^ activates the plasma membrane proton (H^+^)-ATPase in a K^+^-dependent manner. The pattern is quite similar to that in PHE uptake in the presence of K^+^. The external medium pH treated with PHE and K^+^ is higher than that with K^+^, and lower than that with PHE, indicating that H^+^ pump involves in the interaction between PHE and K^+^ uptake.

**Conclusions:**

Therefore, it is concluded that a K^+^ influx/H^+^ efflux reaction is coupled with the transport of PHE into wheat root cells. Our results provide a novel insight into the PHE uptake by crop roots.

## Background

Polycyclic aromatic hydrocarbons (PAHs) are ubiquitous environmental organic pollutants, deriving from natural or anthropogenic activities, especially from incomplete combustion or pyrolysis of organic material [1]. The annual PAH emission in China was estimated up to 114,000 tons in 2004, accounting for 29% of the global total [2]. Due to their carcinogenicity and toxicity to both human and non-human organisms, PAHs have been recognized as priority pollutants by the U.S. Environmental Protection Agency [3]. Dietary intake has been identified as the principal route of exposure to PAHs for the non-smoking population, with plant-based foodstuffs constituting a major contributor to the total PAH intake [4-6]. Furthermore, it has been reported that over 20% of main crops contain PAHs that exceed the relative control limit (5 μg kg^-1^ benzo[a]pyrene) in China [7]. Therefore, it is important to find a way to reduce the uptake of PAHs by crops for food safety.

It has been well documented that plant roots can take up PAHs from soil or water contaminated with PAHs [8-13]. However, the mechanism of PAH uptake by plant roots is still not definitively understood. Our previous work has demonstrated that PAHs can enter the crop roots partly by passive transport and partly by active transport [14]. Passive transport of PAHs proceeds via aquaglyceroporins, and active transport is mediated by H^+^/PAHs symporters [15]. The cotransport of PAHs may be driven by a gradient of electrochemical potential for H^+^ across a membrane, which is generated and maintained by plasma membrane-bound ATPase. Evidence for symport of H^+^/PAHs includes: (a) alkalinization of the medium during PAH uptake; (b) transient depolarization of membrane in response to PAH supply; (c) inhibition of PAH uptake by metabolic inhibitors and ATPase inhibitors; and (d) stimulation of PAH uptake by low external pH [15].

Potassium (K^+^) is classified as an essential macronutrient for all plants. It is the most abundant ion in plant cells and is required for a wide array of functions, such as maintenance of electrical potential gradients across cell membranes, generation of turgor, and activation of numerous enzymes [16]. Hence, it is one of the major fertilizers frequently applied in agriculture.

It is well known that K^+^ uptake is characterized by biphasic uptake kinetics, a low-affinity transport system (LATS) (mechanism 2, i.e., K^+^ channel) and a high-affinity transport system (HATS) (mechanism 1, i.e., K^+^/H^+^ symport) [17,18]. At low external concentrations (under 1 mM), the HATS operates and catalyzes an inward flux, against an electrochemical gradient, by use of a K^+^/H^+^ symport mechanism [19]. By contrast, the LATS dominates mostly via the activity of K^+^ channels at high external concentrations (higher than 1 mM) [20]. K^+^ uptake via HATS or LATS can trigger H^+^ efflux [21,22], resulting in decrease in external medium pH [23,24]. Whether H^+^ efflux associated with K^+^ uptake enhances the H^+^/PAH symport process is unknown.

In this paper, we hypothesize that a K^+^ influx/H^+^ efflux reaction is coupled with the active uptake of PAHs into the root cells. The objectives are 1) to determine the dependence of PAH uptake upon K^+^ influx, and 2) to reveal the mechanism on K^+^-stimulated PAH uptake. The investigation about the relationship of PAH and K^+^ uptake is beneficial for regulating PAH uptake by plant roots through decreased or increased K^+^ fertilizer application in crop food safety and enhanced phytoremediation of PAH-contaminated soils or water. Here, we provide for the first time, to our knowledge, strong evidence of coupling between H^+^/PAH symport and K^+^ uptake.

## Results

### Effect of K^+^ on phenanthrene (PHE) uptake

**Figure 1:**
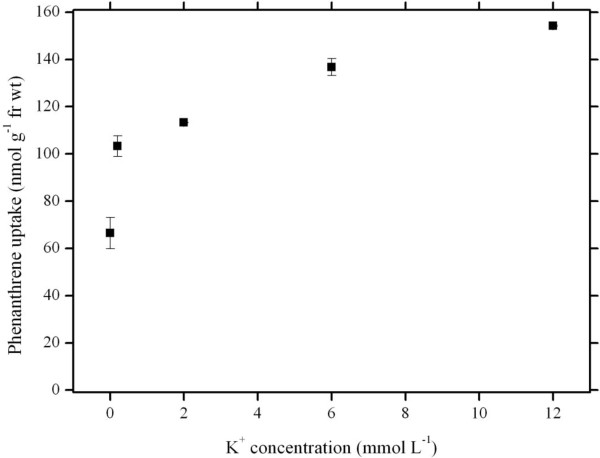
**K**^**+**^**-stimulated phenanthrene uptake by wheat roots.** The uptake of phenanthrene was performed at 25°C for 4 h in modified Hoagland nutrient solutions (i.e., potassium ions were replaced with sodium ions in nutrient solution, pH 5.5) with 5.62 μM phenanthrene. Data points represent mean and SD values of triplicates. Error bars do not extend outside all data points. fr wt, Fresh weight.

To examine the effect of K^+^ on PHE uptake by wheat roots, different levels of K^+^ were added to uptake solutions. Figure 1 depicts the K^+^-dependent PHE uptake. K^+^ noticeably enhanced PHE uptake by roots of intact wheat seedlings in a nonlinear manner within K^+^ concentrations of 0–12 mmol L^-1^ (Duncan’s test, *P* < 0.05). The enhancement was more obvious within K^+^ range of 0–0.2 mmol L^-1^ with a rate (i.e. the increment of PHE uptake divided by the increment of K^+^ concentration) of 184.05 as compared to that at higher K^+^ range of 0.2-12 mmol L^-1^ K^+^ with a rate of 4.34.

### Effect of K^+^ channel inhibitors on PHE uptake

**Figure 2:**
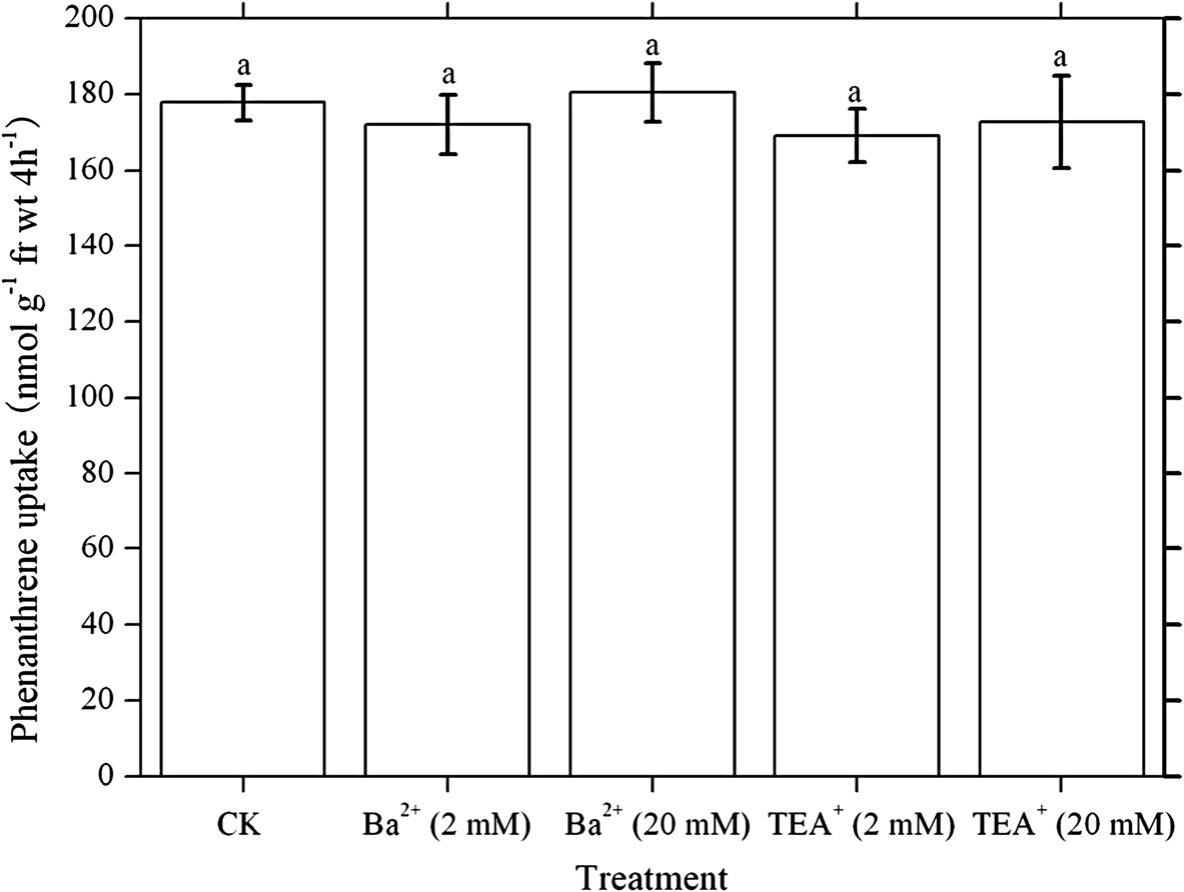
**Uptake of phenanthrene in the presence or absence of blockers (TEA**^**+ **^**and Ba**^**2+**^**) for K**^**+ **^**channels.** Hydroponic solution was Hoagland nutrient solution (pH 5.5) with 5.62 μM phenanthrene. Each histogram bar represents the mean value of triplicates. Bars are the standard deviation of the mean. Data were subjected to one-way ANOVA and compared using the Duncan’s test (*P* < 0.05). The same letters above the error bars indicate no significant differences. fr wt, Fresh weight. TEA^+^, Tetraethlyammonium.

PHE uptake in the presence and absence of Tetraethlyammonium ion (TEA^+^) and Barium ion (Ba^2+^) is shown in Figure 2. Although there existed slight fluctuation among the uptake data, the difference was not significant (Duncan’s test, *P* > 0.05).

### Effect of PHE and K^+^ uptake on membrane potential

**Figure 3:**
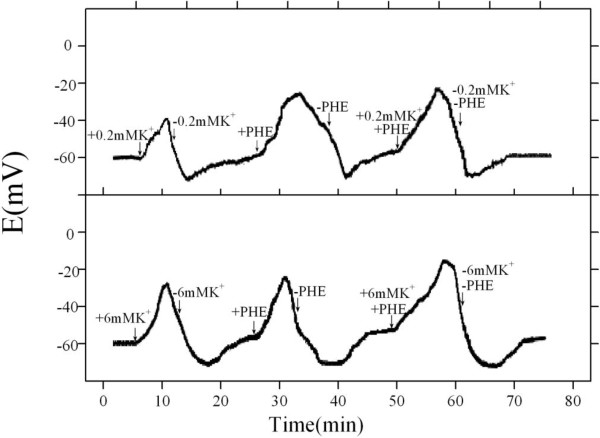
**Phenanthrene-, potassium-, or phenanthrene plus potassium-induced membrane potential changes.** Impalements were made in basal solution (5 mM MES, 0.5 mM CaCl_2_, 0.05 mM NaCl, pH 5.5) with or without 5.62 μM phenanthrene, potassium, or 5.62 μM phenanthrene plus potassium. Wheat roots grew for 15 d. Arrows indicate times at which addition and removal of phenanthrene, K^+^, or phenanthrene and K^+^. MES, 2-(*N*-morpholino)ethanesulfonic acid. PHE, Phenanthrene.

The membrane of wheat root cells depolarized upon introduction of K^+^, PHE, or both K^+^ and PHE (Figure 3). This depolarization was only transient. Depolarization recovered spontaneously while still in the presence of K^+^, PHE, or both K^+^ and PHE. When either K^+^ or PHE, or both K^+^ and PHE were removed from the medium after repolarization completed, the membrane was transiently hyperpolarized and gradually depolarized to values approximately equal to the initial ones (Figure 3). Depolarization, repolarization, hyperpolarization and depolarization could be repeated three times with the same root when it was exposed to K^+^-, PHE-, or K^+^ and PHE-containing solutions and then to K^+^-, PHE-, or K^+^ and PHE-free solutions (data not shown). The depolarization displayed as a function of K^+^ concentration. The higher K^+^ concentration, the higher membrane potential of root cells depolarized (Figure 3). The depolarization elicited by PHE was stronger than by K^+^, and that induced by PHE and K^+^ was greater than that by PHE or K^+^.

### Activity of plasma membrane H^+^-ATPase

**Figure 4:**
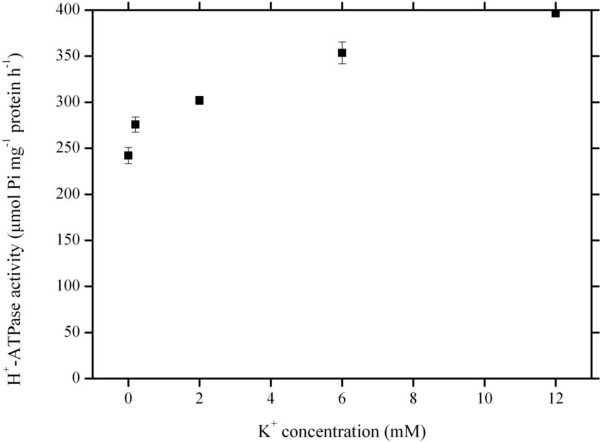
**K**^**+**^**-dependent plasma membrane H**^**+**^**-ATPase activity.** Phenanthrene concentration was 5.62 μM. K^+^ varied from 0 to 12 mM. Data points represent mean and SD values of triplicates. Error bars do not extend outside all data points. Pi, inorganic P.

To characterize the changes in activity of plasma membrane H^+^-ATPase, the enzyme activity was determined at different K^+^ concentrations in the presence of 5.62 μM PHE. Figure 4 shows that the activities of plasma membrane H^+^-ATPase increased as K^+^ concentration increased within the test range of 0–12 mM. The activities increased dramatically at lower K^+^ concentrations up to 0.2 mM; at higher K^+^ concentrations, the activities increased slowly without reaching saturation.

### Effect of K^+^ and PHE uptake on external medium pH

The addition of PHE caused a significant increase in solution pH (Duncan’s test, *P* < 0.05) (Table 1). The uptake of K^+^ markedly reduced solution pH (Duncan’s test, *P* < 0.05), and the higher the K^+^ concentration, the lower the pH was. The pH values treated with K^+^ and PHE were higher than those treated with K^+^ (Duncan’s test, *P* < 0.05), but lower than that treated with PHE (Duncan’s test, *P* < 0.05).

**Table 1:** pH values of hydroponic solution initially and after 4 h of PHE uptake

**Treatment**	**pH**_**1**_	**pH**_**2**_	**ΔpH**
Ck (-K^+^-PHE)	5.50	5.47 ± 0.06d	-0.03 ± 0.06d
+5.62 μM PHE	5.50	5.82 ± 0.03a	0.32 ± 0.03a
+0.2 mM K^+^	5.50	5.38 ± 0.01e	-0.12 ± 0.01e
+0.2 mM K^+^ +5.62 μM PHE	5.50	5.72 ± 0.03b	0.22 ± 0.03b
+6 mM K^+^	5.50	5.32 ± 0.02f	-0.18 ± 0.02f
+6 mM K^+^ +5.62 μM PHE	5.50	5.65 ± 0.02c	0.15 ± 0.02c

Millipore water (pH 5.5) was employed for medium. Data were subjected to one-way ANOVA and compared using the Duncan’s test (*P* < 0.05). Different letters in the same column indicate significant differences. PHE, phenanthrene. pH_1_, pH_initial_. pH_2_, pH_after 4h_. ΔpH = pH_after 4h_ - pH_initial_.

## Discussion

To date, little information is available on the interaction between K^+^ and PAH uptake into plant roots, and the mechanisms underlying K^+^-stimulated PAH uptake remain unclear. Generally, the interaction between K^+^ and PHE uptake may proceed directly by transport pathways or indirectly by altering environmental factors such as external medium pH. If plants take up PHE in the same transport ways as K^+^ (i.e. direct interaction), the presence of K^+^ would inhibit PHE uptake. On the contrary, we have noticed that K^+^ stimulates PHE transport into wheat roots (Figure 1), and the presence of K^+^ channels blockers (TEA^+^ and Ba^2+^) doesn’t repress PHE uptake due to no significant difference in PHE uptake by wheat roots between the treatments with and without TEA^+^ or Ba^2+^ (Figure 2). This means that PHE influx into wheat roots doesn’t proceed via K^+^ transport ways.

It has been well addressed that K^+^ transport into plant roots triggers depolarization in membrane potential [17,25]. And, we have previously reported that a depolarization of membrane potential occurs upon addition of PHE into basal solution, and PHE is taken up by a PHE/H^+^ symport system [15]. The similar phenomena are also found in our electrophysiological measurements (Figure 3). Therefore, the combined treatment of PHE and K^+^ leads to a greater depolarization of membrane potential, especially at higher K^+^ level. Since the active transport for PHE proceeds via a PHE/H^+^ symport system, PHE uptake causes H^+^ entrance into root cells, further triggering a depolarization in root cell membrane potential [15]. Thus, more positive charges pass into root cells in the treatment with both PHE and K^+^ than with PHE or K^+^ only, and then a much greater depolarization of membrane potential occurs in the treatment with PHE and K^+^. The subsequent repolarization in the presence of PHE, K^+^ or both PHE and K^+^ can be explained by increased activity of H^+^ efflux pump stimulated by the increased cytosolic H^+^ concentration [26]. The hyperpolarization upon withdrawal of PHE, K^+^ or both PHE and K^+^ may have been due to the cessation of the solute/H^+^ symport carrier or the closure of K^+^ channels while the H^+^-efflux pump still operates at an increased rate [26].

The plant plasma membrane H^+^-ATPase is very abundant in cells that are active in nutrient acquisition, and plays crucial roles in a number of essential physiological processes [27] such as energization of nutrient uptake, phloem loading, opening of stomata, generation of the electrochemical gradient of H^+^ that provides the driving force for uptake of solutes through channel proteins and H^+^-coupled carriers [28], as well as the regulation of extra- and intracellular pH. Because plasma membrane H^+^-ATPase can be stimulated by K^+^[29], its activity increases with an increase in K^+^ concentrations as shown in our results (Figure 4). Moreover, the pattern of changes in plasma membrane H^+^-ATPase activity in response to increasing K^+^ concentrations is considerably similar to that in PHE uptake (Figure 1). Therefore it is likely that K^+^-stimulated PHE uptake is indirectly coupled with an electrochemical H^+^ gradient established due to the activation of plasma membrane H^+^-ATPase by K^+^ ions, i.e., a K^+^ influx/H^+^ efflux reaction is coupled with the active uptake of PAHs into the root cells.

**Figure 5:**
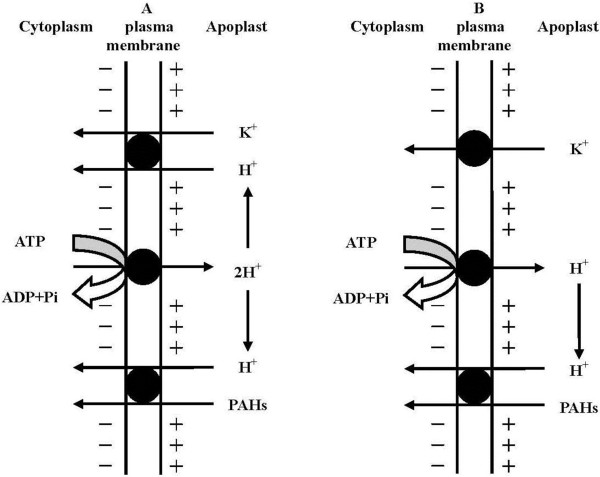
**A conceptual model proposed for the coupling of phenanthrene transport into root cells and a potassium influx/proton efflux reaction. A** is for the high-affinity potassium transport system (HATS), **B** is for the low-affinity potassium transport system (LATS). In HATS, the symport of H^+^/K^+^ activates proton pump mediated by plasma membrane H^+^-ATPase, then forming a transmembrane electrochemical proton gradient. The electrochemical proton gradient drives the phenanthrene/proton symport system. In LATS, K^+^ influx via channels stimulates proton pump, and establishes an electrochemical proton gradient acting as a driving force for H^+^/phenanthrene symport. Pi, inorganic P. PAHs, polycyclic aromatic hydrocarbons.

Increase in K^+^ ions in the hydroponic solution induces a further depolarization of root cell membrane (Figure 3), and thus promotes the release of H^+^ into the external medium, causing a reduction in external medium pH (Table 1). The results are in good line with those observed by Sacchi et al. [30] and Du et al. [24]. At higher K^+^ concentrations, the channel-mediated influx predominates, causing a greater release of H^+^ in the external medium [30]. Therefore, the external medium pH at 6 mM K^+^ is lower than that at 0.2 mM K^+^ (Table 1). Due to an increase in external medium pH for the operation of the H^+^/PHE symport system and a decrease in external medium pH for K^+^ uptake, the external medium pH in the presence of PHE and K^+^ is much higher than that in the presence of K^+^ only (Table 1). The changes in external medium pH during root uptake of PHE with and without K^+^ further support our hypothesis that K^+^-stimulated PHE uptake is coupled with H^+^ pump activated by K^+^. Based on our results and the mechanism for K^+^ transport into plant root cells proposed by Britto and Kronzucker [22], a model for interaction between K^+^ and PHE uptake is proposed (Figure 5). In this model, the H^+^/PHE symport system is associated with a K^+^ influx/H^+^ efflux reaction.

## Conclusions

We have observed the interaction of PHE and K^+^ uptake, and proposed a mechanistic model for the interaction. To our knowledge, this is the first report on the topic. The data presented here show that the PHE uptake is dependent on the external medium K^+^ concentrations. The inhibitors for K^+^ channels have no effect on PHE uptake. The presence of PHE and K^+^ triggers a greater depolarization of root cell membrane as compared to that of PHE or K^+^ only, resulting in a higher external medium pH. The activity of plasma membrane H^+^-ATPase is activated by K^+^. Therefore, the K^+^-enhanced uptake of PHE is a result of a coupling of H^+^-PHE symport into wheat roots and a K^+^ influx/H^+^ efflux reaction. Our results provide a novel insight into the PAH uptake by crop roots.

## Methods

### Chemicals

PHE, a model compound of PAHs [31,32], was purchased from Fluka Chemical Corporation with purity >97%. It has molecular weight of 178.2 g mol^-1^, and water solubility of 7.3 μmol L^-1^at 25°C [33]. All organic solvents used for extraction and analysis of PHE were of high performance liquid chromatography (HPLC) grade.

### Plant germination and cultivation

Wheat (*Triticum aestivum* L.) seeds were surface-sterilized in 10% H_2_O_2_ for 10 min. They were then germinated on moistened filter paper for 4 d at 25°C in the dark after thorough rinsing with Millipore (Milli-Q, Billerica, MA, USA) water. The wheat seedlings were transplanted to black plastic pots containing 2500 mL half-strength aerated Hoagland nutrient solution for 5 d and then transferred to the full-strength Hoagland solution for 5 d. The nutrient solution was prepared with Millipore water and the initial pH of the solution was adjusted to 5.5. Wheat seedlings were grown in a controlled-climate chamber with a light/dark regime of 16/8 h at 25/20°C, a relative humidity of 60%, and a light intensity of 400 μmol m^-2^ s^-1^. After 10-d growth in Hoagland nutrient solution, the wheat seedlings were immersed in Millipore water for 24 h and then employed in the subsequent PHE uptake and electrophysiological study.

### K^+^-dependent uptake of PHE

Twenty intact 15-d-old wheat seedlings with uniform size were transferred to 600-mL beakers containing 500 mL aerated, full-strength, modified Hoagland nutrient solution (i.e., potassium ions were replaced with sodium ions in nutrient solution, pH 5.5) with 5.62 μM PHE and 0.05% methanol as a solvent. In order to improve the dissolution of PHE in nutrient solution, PHE stock solution prepared with methanol as a solvent was added. The methanol concentration was less than 0.1% and had no impact on root growth [34]. The uptake of PHE was detected at 25°C after 4 h of uptake in the modified Hoagland nutrient solution at K^+^ concentrations of 0, 0.2, 2, 6, and 12 mM. There were triplicates per treatment.

### PHE uptake in the presence of K^+^ channel blockers

Although TEA^+^ and Ba^2+^ can suppress the uptake of some cations like Na^+^ and NH_4_^+^[35-37], they are well-known inhibitors of K^+^ channels, and block channel conductance by interacting with sites normally occupied by K^+^ ions [38-40]. Therefore, TEA^+^ and Ba^2+^ were utilized as blockers of K^+^ channel in this study. Each was employed at concentrations of 2 and 20 mM [41] in Hoagland nutrient solution (pH 5.5) with 5.62 μM PHE and 0.05% methanol. The procedures were the same as those in K^+^-dependent uptake of PHE.

### Membrane potential measurements

Wheat (15 d old) root tip was excised, and mounted in a Plexiglas chamber attached to the stage of an Olympus compound microscope, which was fixed to the surface of a vibration-damped table (Kinetic Systems Inc.). The Plexiglas chamber was perfused with basal solution (5 mM 2-(*N*-morpholino) ethanesulfonic acid (MES), 0.5 mM CaCl_2_, 0.05 mM NaCl, 0.05% methanol, pH 5.5) at a flow rate of 10 mL min^-1^ for 2 h before the measurements. Impalement of micropipette into root epidermal cells was made in a region about 1 to 2 cm from the root apex, using a hydraulically driven Narashige micromanipulator mounted on the microscope stage [42,43]. Micropipettes with a tip diameter of <0.5 μm were pulled from filament-containing borosilicate glass capillaries (Clark, GC 150 F) with a vertical puller (PE-21, Narishige Scientific Instrument Lab, Japan). The micropipettes were filled with 0.1 M KCl, and the reference salt bridge with 0.1 M KCl in 2% agar. They were connected by Ag/AgCl electrodes to a WPI amplifier, model FD223. The reference electrode was kept in the perfusion chamber in the vicinity to the root. Measured membrane potentials of root cells, which are the voltage differences between the impaling and reference electrodes, were amplified and recorded on a strip chart recorder. When the resting potential (measured in basal solution) was constant, basal solution was replaced by test solution. The measurements were conducted in the dark at room temperature (about 25°C).

### Determination of plasma membrane H^+^-ATPase activity

Plasma membrane vesicles were isolated according to Yan et al. [44]. Plasma membrane H^+^-ATPase activity was determined as described by Liang et al. [45] with some modifications. Isolated vesicles of plasma membrane (50 μL) were added into 450 μL of reaction medium, containing 30 mM *N*-2-hydroxyethyl piperazine-*N*′-2-ethanesulfonic acid (Hepes)–hydroxymethyl aminomethane (Tris) (pH 6.5), 50 mM NaNO_3_, 3 mM MgSO_4_, 0.1 mM ammonium molybdate, 2 mM ATP-Na_2_, 5.62 μM PHE and 0.05% methanol, at K^+^ concentrations of 0, 0.2, 2, 6, and 12 mM. After 20 min of incubation at 37°C, reaction was terminated by adding 50 μL of trichloroacetic acid. Inorganic phosphate (Pi) released from ATP hydrolysis was determined by the colorimetric molybdenum blue method. Protein in membrane vesicles was analyzed colorimetrically at 595 nm based on the formation of protein–dye complex (the binding of Coomassie Brilliant blue G-250 to protein) using bovine serum albumin as the standard. Plasma membrane H^+^-ATPase activity is expressed as μmol Pi mg^-1^ protein h^-1^.

### Change in incubation solution pH

External medium pH alteration depends mainly on H^+^ efflux caused by the activity of plasma membrane H^+^-ATPase and on H^+^ influx from cotransport activity. To facilitate the detection of pH changes in K^+^-, PHE-, or K^+^and PHE- incubation solutions, the contribution of other cations and anions was minimized. For this purpose, the experiments were performed in Millipore water. Wheat roots were immersed in six sorts of incubation solution with initial pH of 5.5: (1) Millipore water, (2) Millipore water with 5.62 μM PHE, (3) Millipore water with 0.2 mM K^+^, (4) Millipore water with 0.2 mM K^+^ and 5.62 μM PHE, (5) Millipore water with 6 mM K^+^, and (6) Millipore water with 6 mM K^+^ and 5.62 μM PHE. After 4 h of incubation, pH values of incubation solution were measured with a pH meter. The experiment was performed as described in K^+^-dependent uptake of PHE.

### Extraction and analysis of PHE

The PHE in wheat was extracted and detected using the method of Zhan et al. [14]. After harvest, wheat roots were immersed in methanol for 3 min, and then rinsed with sufficient Millipore water to remove the PHE on root surface, followed by wiping with tissue paper [46,47]. Wheat roots and shoots were weighed and ground in a glass homogenizer. Homogenized tissue samples were extracted with acetone/hexane (1:1, v/v) mixture by ultrasonication three times (30 min each time). The combined extracts were passed through an anhydrous Na_2_SO_4_ column with elution of the 1:1 mixture of acetone and hexane. The eluents were then evaporated to dryness at 35°C in a rotary evaporator and dissolved in 12 mL hexane. Subsequently, the 12-mL solvent was cleaned in a 2-g silica gel column and eluted with 25 mL hexane/dichloromethane (1:1, v/v) solvents. The eluents were evaporated to dryness again and dissolved in 2 mL methanol. Prior to the analysis of PHE by HPLC, all final extracts were filtered with 0.22 μm filter [8]. The average recovery of PHE acquired by spiking wheat samples with standards is 95.2% for the entire procedure. None of the data reported here has been corrected for recovery.

The HPLC system employed consists of an automatic injector (Waters 717), a binary high-pressure pump (Waters 1525), a UV detector (Waters 2487), and a fluorescence detector (Waters 2475). Separations were performed with a reverse phase column (Waters Symmetry C_18_ column, 5 μm, 4.6 × 150 mm). The temperature of the HPLC column was kept constant at 30°C. The mobile phase was methanol/Millipore water (80:20, v/v), with a flow rate of 1 mL min^-1^. The injection volume was 10 μL. PHE was quantified at 293.5/395 nm (excitation/emission wavelength) and 254 nm for fluorescence and UV detector, respectively. Relative standard deviation (n = 5) was less than 2.85% for the method. The method detection limit was 48.5 pg PHE. Analytical standards were measured at the beginning of each series of analyses. Internal standard calibration was performed during the HPLC analyses.

### Statistical analyses

Statistical analyses were performed with SAS software version 9.1.3 (SAS Institute Inc., Cary, NC, USA). PAH contents in wheat tissues and pH values of nutrient solution were subjected to one-way analysis of variance (ANOVA) and compared using Duncan’s test at *P* < 0.05.

## Abbreviations

ANOVA: Analysis of variance; Ba2+: Barium ion; fr wt: Fresh weight; H+: Proton; HATS: High-affinity transport system; HPLC: High performance liquid chromatography; K+: Potassium ion; LATS: Low-affinity transport system; MES: 2-(*N*-morpholino)ethanesulfonic acid; PAHs: Polycyclic aromatic hydrocarbons; PHE: Phenanthrene; Pi: Inorganic P; SD: Standard deviation; TEA+: Tetraethlyammonium ion.

## Competing interests

The authors declare that they have no competing interests.

## Authors’ contributions

XZ, TJ and GX designed research; XZ and XL performed research; XZ, XL, TJ and GX analyzed data; and XZ, TJ and GX wrote the paper. All authors read and approved the final manuscript.
